# A comparison of maternal and newborn health services costs in Sindh Pakistan

**DOI:** 10.1371/journal.pone.0208299

**Published:** 2018-12-06

**Authors:** Asif Raza Khowaja, Craig Mitton, Rahat Qureshi, Stirling Bryan, Laura A. Magee, Peter von Dadelszen, Zulfiqar A. Bhutta

**Affiliations:** 1 Department of Obstetrics and Gynaecology, University of British Columbia, Vancouver, Canada; 2 Centre for Clinical Epidemiology and Evaluation and School of Population and Public Health, University of British Columbia, Vancouver, Canada; 3 Division of Women & Child Health, Aga Khan University, Karachi, Pakistan; 4 Department of Women and Children's Health, School of Life Course Sciences, Faculty of Life Sciences and Medicine King's College London, United Kingdom; 5 Program for Global Pediatric Research, Hospital For Sick Children, Toronto, Canada; Navrongo Health Research Centre, GHANA

## Abstract

Pakistani women suffer the highest rate of maternal mortality in South Asia. A lack of comprehensive knowledge about maternal and newborn health (MNH) services costs impedes policy decisions to maximize the benefit from existing, as well as emerging, MNH interventions in Pakistan. We compared MNH service costs at different levels of care. A cross-sectional survey was conducted during January to March 2016 as part of a large economic evaluation in Sindh, Pakistan. Health providers and facilities were selected from a sampling frame, inclusive of public and private sectors. This study utilized a broad perspective (i.e. costs to the health system and patients/families). The unit costs of MNH services were determined through a simultaneous allocation method in the public facilities; and patient billing department in the private facilities. Descriptive analysis was performed, and an analysis of variance (ANOVA) test was applied to compare overall mean costs both within and between health facilities. A total of 31 eligible health providers and facilities (n = 25 in private; n = 7 in public) were included in the final analysis. An ambulatory visit (AV) for routine antenatal care (ANC) costs $3.6 and $0.9 at secondary- and tertiary-level public facilities, respectively. In the private sector, the costs of an AV for ANC were slightly less ($2.8) at secondary-level and much higher ($6) at tertiary-level facilities compared to the public sector. Diagnostic test costs were much higher in private facilities. The average costs of inpatient admissions were $30.5 at general ward (GW), and $151 at the intensive care unit (ICU) in public facilities. In-patient admissions costs were lower such as $9.3 at GW and $36.5 at ICU in private facilities. Understanding cost is critical to guide decisions of resource allocation within the public sector; and risk mitigation for excessive OOP costs through third party payer for services in the private sector.

## Introduction

Globally, maternal and newborn mortality has declined over the past 10 years [1]. The 2015 estimates from the Global Burden of Disease indicate that most countries (122 of 195, 63%) have achieved Sustainable Development Goal 3.1, a reduction of global maternal mortality ratio to less than 70 per 100,000 live births by 2030 [2]. However, high burdens of both maternal and newborn mortality continue to impose a significant challenge in many low- and middle-income countries (LMICs), where resources to seek and provide timely and effective healthcare are scarce [3].

Pakistan has the world’s sixth largest population and has the highest maternal mortality ratio in South Asia (348 per 100,000 live births). This compares poorly with neighboring countries, including Bangladesh and Bhutan, and many countries in sub-Saharan Africa where maternal deaths have substantially declined since 1990 [2]. In Pakistan, a situational analysis revealed that only 65% pregnant women seek routine antenatal care (ANC), nearly 48% deliveries occur without the assistance of a skilled care provider, fewer than 50% of women seek either postpartum and/or newborn care [4]. In the community, the Lady Health Workers (LHWs) under the National Program provide basic health education related to antenatal, post-partum, and newborn care; and serve as a referral point to health facilities. In the community, patients (sick mothers and/newborns) often skip the referral sequence and present directly to a higher facility (i.e. a tertiary-level) given concerns about the sub-optimal quality of care and poor staffing at the lower levels [5]. At a tertiary-level health facility, a comprehensive ANC is provided by the medical doctors specialized in the obstetrics and gynecology. The continuity of care, however, is mainly influenced by individuals’ ability-to-pay, geographical access, and availability of transport [6]. Data related to health expenditures in Pakistan revealed a declining trend of gross domestic product (GDP) spending on health over the past decade; currently as low as 2.4%. It is further estimated that over 80% of healthcare spending is out-of-pocket (OOP), and predominately in the private sector [7].

Given the rising costs of care related to pregnancy and childbirth, health policy/decision makers are keen to explore innovative solutions through health technology (HT) integration in the area of maternal and newborn health (MNH) [8–9]. Currently, mobile health (mHealth) technologies are used for early detection of diseases during pregnancy, and child vaccination reminders in Pakistan [10–11]. In an earlier study, short message services (SMS) and cell phone reminders were associated with significantly higher rates for clinic attendance and treatment adherence for tuberculosis [12]. This shift towards the beneficial use of HT integration has implications for incremental costs to patients, health systems and society at large [13]. However, a lack of compressive knowledge of the cost of MNH services at different levels of care confounds policy decisions about introducing existing interventions and impedes economic appraisal of emerging HT in Pakistan.

The Community-Level Interventions for Pre-eclampsia (CLIP) cluster randomized controlled trial is testing an innovative package of care that introduces mHealth platform-guided case identification, time-of-disease risk stratification, and case-management for women with a hypertensive disorder of pregnancy (HDP) in Pakistan, India, and Mozambique [10]. The assessment of cost-effectiveness of the CLIP trials requires a thorough understanding of maternal and newborn costs at health facilities in the CLIP countries. In designing an economic model for the CLIP trial in Pakistan, similar challenges were faced, as health facility costs were unknown for care received during pregnancy, delivery and early newborn stages in both private and public sectors. The primary objective of this study was to estimate the cost of MNH services. The secondary objective was to compare the cost of MNH services within and/or between public and private health sectors in Pakistan.

## Materials and methods

### Study design

A cross-sectional survey of health facilities was conducted during January to March 2016, as part of a large economic evaluation of the CLIP Trial in Pakistan. This study utilized a broad perspective (i.e. costs to the health system and patients/families). The details on the methods and perspective for the economic evaluation are described elsewhere [14].

### Study settings

This study was conducted in two neighboring districts, Matiari and Hyderabad, located in the southern province of Sindh, Pakistan. The provision of basic, as well as comprehensive, emergency obstetric and newborn care (EmONC) services, were available in the private health sector (i.e. 100% OOP costs to patients/families). In the public sector, the MNH services are not entirely free- and require some OOP contributions from patients/families (selected medications, food, and transport); and user-fees for diagnostic tests. Health facilities were clustered into three broad categories: primary, secondary and tertiary levels. Categorization was based on the population served, hospital size (usually, number of beds), and the provision of clinical subspecialty services and intensive care. The primary level health facilities provide health services to less than 50,000 people, with an inpatient capacity of 0 to 10 beds, and focus on basic obstetric and newborn care. The secondary level hospitals provide health services to over 1 million people, with an inpatient capacity of 40 to 60 beds, and focus on basic and EmONC services. The tertiary level hospitals serve as the referral point, provide multispecialty clinical services, and offer intensive care facilities to a wider population [15].

### Inclusion and exclusion criteria

**Fig 1:**
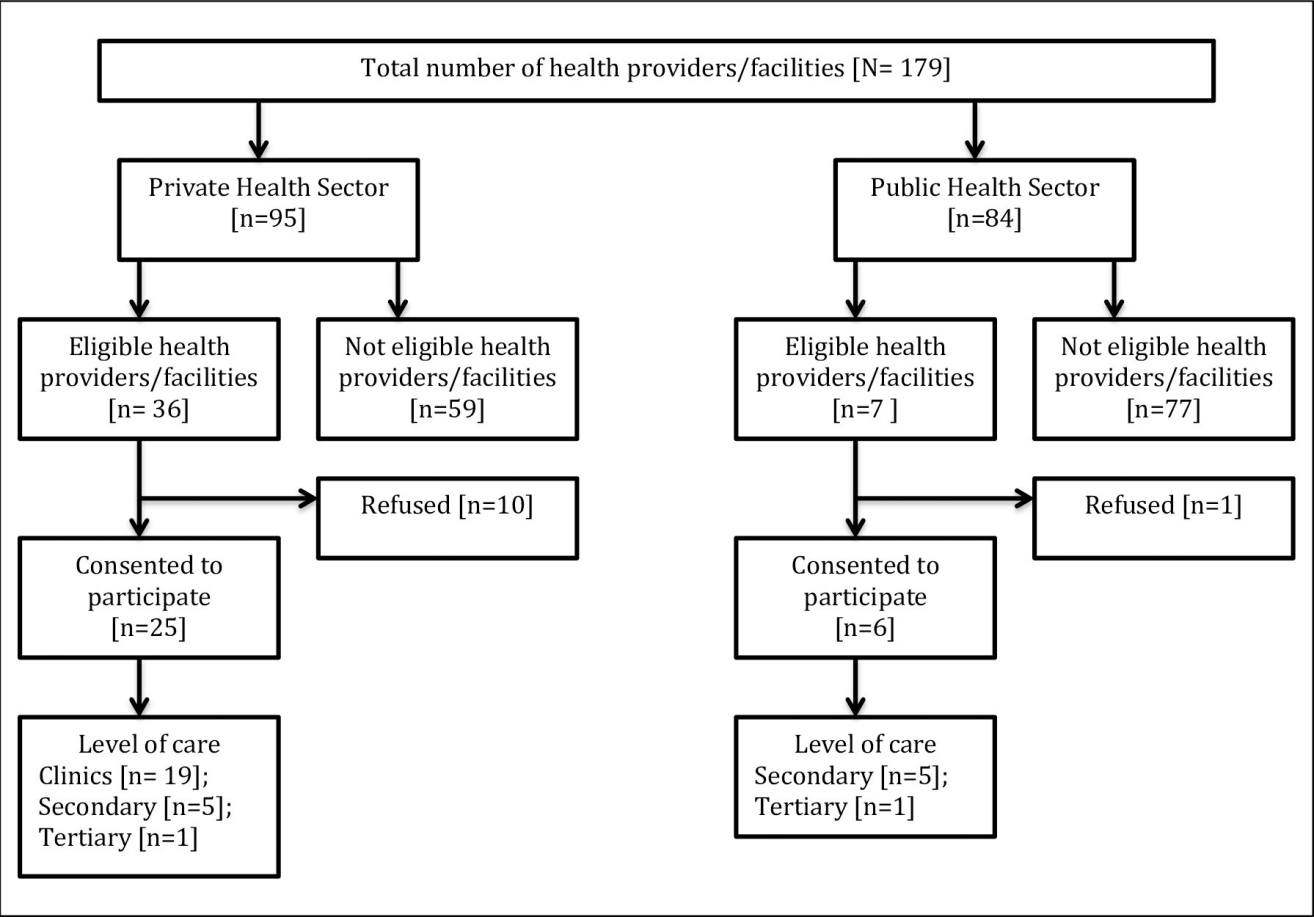
Health care providers and facilities in the public and private health sector.

The healthcare providers and facilities were considered eligible if they met these criteria: (i) geographical location within study catchments; (ii) secondary and tertiary level hospitals in the public health sector, to which pregnant women are referred by LHWs under the National Program; and (iii) private healthcare providers and facilities, where pregnant women self-refer for pregnancy care and childbirth. Primary level facilities in the public health sector; and healthcare providers and facilities that declined to participate were excluded (Fig 1).

### Sampling procedures

Healthcare providers and facilities were selected from a sampling frame, inclusive of the public and private health sector in the study catchments, as previously described [16]. A list of public health facilities was obtained from the office of district health officer (DHO) and referral health facilities (i.e. secondary and tertiary-level facilities) were identified through the National LHWs Program. Private healthcare providers were identified through the CLIP Pakistan trial network, and health facility mapping work from previous MNH research projects led by the Aga Khan University, Karachi Pakistan. The project field coordinator approached the administrative staff at health facilities and invited them to participate in the study.

### Methods of data collection

This study evaluated costs of health facility resource utilization for acute illnesses during pregnancy and/or newborn period (i.e. < 1 year). A structured questionnaire was used to collect cost data of MNH services. The key variables included: ambulatory visits (AV) for routine antenatal and newborn care, diagnostic tests and imaging, overnight inpatient admission, childbirth, and blood transfusion. Project research assistants (RAs; registered midwives or bachelor’s degree holders with MNH research experience) were trained by a Senior Scientist, who was a native speaker of the local Sindhi language and had experience in MNH research in Sindh.

RAs visited health care providers and facilities and ascertained unit costs from billing departments as they were charged to patients in the form of fee-for-service at the private facilities. In public sector, costs were obtained from multiple sources including administrative/financial records, inventory audits of in-patient areas, staff register, and consultation with the hospital administrative staff (e.g. Medical Director, Unit Registrar, and Finance Manager) at respective sites. Capital costs comprised of clinical equipment, air-condition, power-generator, computers, and furniture and fixture. Recurrent costs included medications and clinical supplies. Other recurrent costs were shared between departments (i.e. staffing, utility, laundry, housekeeping, repair-and-maintenance, and patient food). A simultaneous allocation method was used to estimate the unit costs of interdepartmental services. This approach is also known as a reciprocal method that uses simultaneous equations to provide a more accurate allocation of service department costs in a given proportion [17]. The department-level costs were later divided by the average number of patients attending clinics and the number of beds to calculate the unit costs for ambulatory visits and inpatient admissions, respectively. The costs were estimated in the local currency, Pakistani Rupee (PKR), and later converted into US$ [$1 US$ = 104.7 PKR; 25 Oct 2016].

### Data analysis

Descriptive analyses were performed to calculate mean and standard deviations for cost estimates from public and private hospitals, except tertiary level hospitals where point-estimates were reported. The analyses of variance (ANOVA) tests compared overall mean costs within and between public and private health sectors, and statistically significant differences were interpreted with a p-value (two-tailed) <0.05. Analyses were performed using SPSS version 24.

### Ethical considerations

This study received ethical approval from the Ethics Review Committee (ERC) of Aga Khan University located in Karachi, Pakistan (1917-OBS-ERC-11), and the Institutional Review Board (IRB) from the University of British Columbia in Vancouver, Canada (H12-00132). Formal health system approvals were obtained at both provincial, as well as district-level, health departments. Participation in this study was voluntary, and written informed consent were acquired from individual health care providers and facilities prior to data collection.

## Results

A total of 43 eligible health care providers and facilities (n = 36 private; n = 7 public) were approached within the study catchments. The refusal rate varied from 14% (n = 1/7) to 27.8% (n = 10/36) in the public and private health sectors, respectively. The final cost analysis included 25 facilities in the private health sector (19 primary-, five secondary- and one tertiary-level facilities), and six facilities in the public health sector (five secondary- and one tertiary-level facilities). The public facilities were geographically scattered, whereas private healthcare providers and facilities were in close proximity.

### Cost of maternal health services

An AV for routine ANC from a medical doctor costs $3.6 (±SD 2.1) at the secondary level; and $0.9 at tertiary level public facilities. In the private health sector, the mean cost of AV for routine ANC were $2.2 (±1.9) in medical clinics, $2.8 (±2.6) in the secondary level, and ($6) in tertiary level facilities. The AV was less costly for routine ANC provided by midwives ($0.6 ± 0.3) and traditional birth attendants ($0.5 ± 0). The costs of many diagnostic tests or imaging were similar within all levels of public facilities, such as pregnancy ultrasound ($0.5 per scan), Creatinine ($0.4 per test), Urine microscopy (0.3 per specimen) and CT scan ($15 per scan). In the private health sector, costs of diagnostic tests were much higher at the tertiary level. For instance, the diagnostic costs in the private tertiary-level facility were $6, $4.3, $2.6, $20 for Pregnancy ultrasound, Creatinine, Urine microscopy, and CT scan, respectively.

The cost of inpatient general ward admission was higher in the tertiary level, compared with secondary-level public facilities ($57.5 vs 3.4 ± 1.7). The costs of delivery were low at secondary level facilities for spontaneous childbirth ($45.4 ±SD 30.7 vs 79), and assisted childbirth, compared with the tertiary level public facility ($50.2 ±SD 33.8 vs 86), respectively. The cost of Caesarean delivery was less in the public tertiary facility, compared with the private tertiary facility ($223.8 vs 400, respectively). Significant differences were found in the overall mean costs for maternal health services within/between public and private facilities at p-values < 0.05 (Table 1).

**Table 1:** Cost of maternal health services in public and private health sectors.

Variables	Public Health Sector Costs (US$)			Private Health Sector Out-of-pocket Costs (US$)				Mean Difference (Overall private minus public)	*p-*value
	Secondary hospitals (n = 5) Mean (SD)	Tertiary hospital* (n = 1)	Overall public (n = 6) Mean (SD)	Clinics/care provider (n = 19) Mean (SD)	Secondary hospitals (n = 5) Mean (SD)	Tertiary hospital* (n = 1)	Overall private (n = 25) Mean (SD)		
**Ambulatory visits for routine antenatal care (cost per visit)**									
Medical doctor	3.6 (2.1)	0.9	2.3 (2.1)	2.2 (1.9)	2.8 (2.6)	6	3.7 (2.2)	1.4	<0.0001
Nurse	NA	NA	-	1.7 (2.1)	NA	NA	1.7 (2.1)	1.7	
Midwife	NA	NA	-	0.6 (0.3)	NA	NA	0.6 (0.3)	0.6	
Traditional birth attendant	NA	NA	-	0.5 (0)	NA	NA	0.5 (0)	0.5	
**Diagnostic tests (cost per test)**									
Pregnancy ultrasound	0.5 (0)	0.5	0.5 (0)	2.8 (1.1)	3.9 (3.2)	6	4.2 (2.4)	3.7	<0.0001
Complete blood count	0.9 (0.9)	0.4	0.7 (0.6)	2 (1.4)	3.8 (2.2)	5.2	3.7 (2.2)	3	
Culture	1.3 (1.1)	0.6	0.9 (0.8)	NA	6.9 (6.6)	4.2	5.6 (4.9)	4.7	
Creatinine	0.4 (0.2)	0.4	0.4 (0.1)	NA	3.4 (2.8)	4.3	3.9 (2.2)	3.5	
Serum albumin	0.4 (0.2)	0.2	0.3 (0.1)	NA	3.2 (0.8)	3.2	3.2 (0.8)	2.9	
Aspartate Aminotransferase	NA	NA	-	NA	6	NA	6	6	
Alanine aminotransferase	NA	0.8	0.8	NA	3.8 (2.7)	1.9	2.9 (1.3)	2.1	
Urine dipstick	0.3 (0)	NA	0.3 (0)	0.8 (0.5)	1.3 (1.2)	NA	1.1 (0.9)	0.8	
Urine microscopy	0.3 (0)	0.2	0.3 (0.1)	NA	1.8 (1.5)	2.6	2.2 (1.4)	1.9	
Chest x-ray	0.9 (0.6)	0.5	0.7 (0.3)	NA	2.5 (1.4)	6	4.3 (2.1)	3.6	
CT scan	NA	15	15	NA	NA	20	20	5	
**In-patient admissions (cost per overnight stay)**									
General ward	3.4 (1.7)	57.5	30.5 (38.3)	4.2 (1.4)	8.5 (11.1)	15.2	9.3 (8.7)	-21.2	0.0136
Intensive care	NA	151.2	151.2	NA	NA	36.5	36.5	-114.7	
**Delivery and blood transfusion (cost per procedure)**									
Spontaneous vaginal delivery	45.4 (30.7)	79.1	62.3 (30.7)	33.3 (14.8)	86 (92.4)	150	89.8 (93.1)	27.5	<0.0001
Assisted vaginal delivery	50.2 (33.8)	86.6	68.4 (33.8)	35.8 (23.7)	106 (108.8)	200	113.9 (104.6)	45.5	
C-section	NA	223.8	223.8	NA	304 (167.2)	400	352 (154.3)	128.2	
Blood transfusion	NA	20.1	20.1	NA	17.8 (22.3)	25.8	21.8 (19.6)	1.7	

* Mean and standard deviation not calculated for one facility; SD: Standard deviation; NA: not available at designated health facility

### Cost of newborn health services

The mean cost of newborn AV in the secondary-level hospitals was $2.7 ± 1.8 compared with $0.4 at the public sector tertiary-level hospital. Newborn AV costs were lowest ($0.5 ± 0) when provided by the midwife and traditional birth attendants in the private health sector. Overall, the cost of fetal ultrasound ($0.5 per scan in public, versus $5.4 per scan in private), newborn x-ray ($0.5 per imaging in public, versus $3.8 per imaging in private), and blood grouping ($0.3 per specimen in public, versus $2.8 per specimen in private) differed substantially at all levels of health facilities in public and private sectors. Phototherapy was only available at tertiary-level facilities, and cost ranged from $1 to 2 in the public and private sectors, respectively. The cost of newborn admission into a nursery was low in the public compared with private tertiary-level facilities ($10.3 vs 13.8 ± 3.7). However, the cost of newborn intensive care admission was higher in the public compared with private tertiary-level facilities ($22.3 vs $19 ± 7.8). Significant differences were found in the overall mean costs for newborn health services within/between public and private facilities (Table 2).

**Table 2:** Cost of newborn health services in public and private health sectors.

Variables	Public Health Sector Costs (US$)			Private Health Sector Out-of-pocket Costs (US$)					Mean Difference (Overall private minus public)	*p-*value
	Secondary hospitals (n = 5) Mean (SD)	Tertiary hospital* (n = 1)	Overall public (n = 6) Mean (SD)	Clinics/care provider (n = 19) Mean (SD)	Secondary hospitals (n = 5) Mean (SD)	Tertiary hospital* (n = 1)	Overall private (n = 25) Mean (SD)			
**Ambulatory visits for newborn care (cost per visit)**										
Medical doctor	2.7 (1.8)	0.4	1.6 (1.7)	1(0)	2.6 (1.1)	2	1.9 (1.1)	0.3		0.0001
Nurse	NA	NA	-	2.3 (2.5)	NA	NA	2.3 (2.5)	2.3		
Midwife	NA	NA	-	0.5 (0)	NA	NA	0.5 (0)	0.5		
Traditional birth attendant	NA	NA	-	0.5 (0)	NA	NA	0.5 (0)	0.5		
**Diagnostic tests (cost per test)**										
Fetal ultrasound	0.5 (0)	0.5	0.5 (0)	4 (0)	6.3 (6.4)	6	5.4 (4.7)	4.9		<0.0001
Fetal x-ray	1 (0)	0.5	0.5 (0.3)	1.5 (0)	3.8 (2.3)	6	3.8 (2.4)	3.3		
Blood grouping	0.3 (0)	0.2	0.3 (0.1)	NA	2.2 (2.8)	3.4	2.8 (2.3)	2.5		
Arterial blood gases	NA	NA	-	NA	12.9 (0)	8.5	10.7 (3.1)	10.7		
Phototherapy	NA	1	1	NA	NA	2	2	1		
**In-patient admissions (cost per overnight stay)**										
Nursery	NA	10.3	10.3	NA	11.2 (0)	16.4	13.8 (3.7)	3.5		0.0011
General ward	4.5 (0)	8.2	6.4 (2.6)	NA	5.9 (1.28)	12.1	9 (3.6)	2.6		
Intensive care	NA	22.3	22.3	NA	17.8 (10.6)	20.2	19 (7.8)	-3.3		

* Mean and standard deviation not calculated for one facility; SD: Standard deviation; NA: not available at designated health facility

## Discussion

This study reports the costs of a wide range of MNH services relevant to families (i.e. OOP), as well as health systems and has demonstrated that these costs vary significantly at different levels of health facilities within and between public and private sectors in Sindh, Pakistan. Our findings provide a robust measure of unit-costs by service type and levels of health facility for future economic studies aiming to estimate cost-of-illness in the area of maternal and newborn care in Sindh, Pakistan.

PE/E are serious conditions during pregnancy requiring frequent AVs for routine antenatal care, diagnostic tests, and health facility admissions in the event of disease complications. The costs of health services at private health facilities as reported in this study suggest a large financial burden related to OOP payments on families. We found a progressive trend in the costs for AV dependent on the level of private healthcare (i.e. costs lower at medical clinics and higher at the tertiary level). Similarly, the costs of diagnostic tests and imaging were higher in private facilities. A previous study evaluated the role of public spending on health care across 11 Asian countries. Authors in that study found that distribution of public health infrastructure is mainly biased towards the provision of services for the wealthy (pro-rich) in many LMICs, and that transition towards pro-poor healthcare requires limiting user fees, protecting the poor from catastrophic expenditure on health, and creating a wide network of public health facilities [18].

The private sector is expanding within the health industry in LMICs and employs social marketing techniques to attract patient volume [19]. In a previous qualitative study, authors found that many women sought pregnancy-related care from private health facilities [7]. Despite higher costs, decisions to seek care are often dominated by the perception of quality of care at such facilities [20]. In another study, the authors assessed patients’ perception of service quality in Pakistan and reported that private health facilities deliver a better quality of care to patients compared with public facilities [21]. Guided by our local field observations, we determined that the private health sector tends to offer cutting-edge diagnostic technologies (i.e. latest and/or expensive imported machines from abroad) to meet the growing demand for precision medicine in Pakistan. We assume that the private health sector could offer lower inpatient prices possibly through cost containment (i.e. controlling overhead expenditures, rational distribution of lower- and higher- cadre of medical staff based on patient volume), and operating with low-profit margins to attract greater patient volumes. More efforts are needed to regulate the private sector and promote strategic purchasing to be able to lower health services costs. This study does not estimate profit margins on the cost of MNH services in the private sector.

Our findings further highlight the policy implications for health systems, as AV for maternal and newborn care are more costly at the secondary-, compared with tertiary-, level public facilities in Pakistan. These findings are similar to those observed in the Northern Province of Pakistan, where AV costs were higher (i.e. $4.1) at basic health units, and reflect ambulatory costs being mainly dependent upon the number of patients attending outpatient clinics (i.e., fewer patients, more cost per AV) [22]. Upon reviewing records of the patient registry at secondary-level facilities, we found that fewer pregnant women were attending outpatient clinics (range: 25 to 50 per day), compared with 250 to 500 per day) at the tertiary level public facility. Previous studies indicated several factors such as inadequate staffing, poor facilities for in-patient admissions, and a poorly-coordinated referral system are responsible for low patient-volumes at lower-level health facilities [23–24]. Furthermore, we found that costs of inpatient admissions, other than for delivery, were much higher in the public facilities. The cost allocation exercise indicated higher operating costs (i.e., staff salaries, equipment, medication, and overheads) at inpatient departments. Others also reported increased cost burden in public health facilities, assuming that patients are presenting with severe illnesses and/or disease complications (requiring longer inpatient stays) as a result of delaying care [25].

The economic appraisal of emerging HT requires robust data on costs and health outcomes using a societal perspective, inclusive of care receivers, care providers and the health system [26]. Our findings are critical to calculating real-world cost-inputs representing public and private health sectors, as we embark upon analyzing MNH resource utilization (end-user data) reported from the intervention and control groups in the CLIP Trial in Pakistan. In addition, the methodological approaches and our findings may guide future health economics studies evaluating MNH interventions in Pakistan and other LMICs.

### Strengths and limitations

To our knowledge, this is the first study to estimate the cost of MNH services at different levels of health facilities in the public and private sectors in Pakistan. We benefitted from high participation (86%) of public secondary- and tertiary-level facilities. The primary-level health facilities were omitted from our analysis because they did not serve as a referral point for EmONC services in the community. Also, primary health facilities were large in numbers, which would have required additional project expenses to capture all possible facilities. We recognize that costing information from primary-level facilities may have been important for comparative analysis across three tiers of the public health system in Pakistan and it is the main limitation of the current study. The capital costs were not discounted, and it is another limitation of this study.

Collecting evidence-based information on costs and health outcomes is often challenging in the absence of electronic medical records and poor data keeping in LMICS [27]. We faced similar challenges in the public and private health sectors. It was a resource-intensive exercise, requiring frequent long-distance travel to public hospitals and several in-person meetings with people in the clinical, budget and finance departments. In the private health sector, people were reluctant to share cost information on MNH services and hospital budgets (i.e. revenue and expenses) due to fear of litigation. Also, some private health facilities were only open in the evening, which created operational difficulties for the project RAs and data collection. We encountered a high refusal rate in the private health sector because of operational difficulties and reluctance to share costing information with persons other than patients. We increased the sample size particularly in the private sector by three-fold, compared to the public health sector, to address the possibility of non-response bias in data collection.

## Conclusions

This economic appraisal of MNH services revealed cost disparities within the public health sector suggesting higher costs for AV at the secondary-level, and inpatient admissions at tertiary-level health facilities in Sindh, Pakistan. The private sector stands-out as an expensive choice of care provider for diagnostics and delivery. An understanding of MNH costs is critical to guide resource allocation within the public sector and for risk mitigation against excessive OOP costs through third-party payer for services in the private sector.
